# Sunk costs account for rats’ decisions on an intertemporal foraging task

**DOI:** 10.1186/1471-2202-13-S1-P63

**Published:** 2012-07-16

**Authors:** Andrew M Wikenheiser, A David Redish

**Affiliations:** 1 Graduate Program in Neuroscience, University of Minnesota, Minneapolis, MN 55455, USA; 2 Department of Neuroscience, University of Minnesota, Minneapolis, MN, 55455, USA

## 

Decision makers commit the sunk cost fallacy when they are influenced by previous investments instead of estimated future returns [1]. As resources previously allocated toward an outcome are irrecoverable, adaptive choices should be based only on the future benefits that could result from available options. Humans clearly show sensitivity to sunk costs in a wide range of situations [1]; however, there is at best mixed evidence that other species are affected by sunk costs, leading to the suggestion that sunk cost sensitivity depends on complicated cognitive/meta-cognitive mechanisms unlikely to be present in non-human animals [2].

We tested the extent to which rats are influenced by previous investment and expected future gains in a naturalistic foraging context. Rats foraged for food on a circular path outfitted with three equidistant feeder sites. Each feeder was associated with a delay that remained fixed within a session. Six sets of delays were chosen to create six session types, across which the opportunity cost of time [3] varied. Upon approaching a feeder, subjects made a stay/go decision; if they remained until the delay expired, food pellets were dispensed. Otherwise, they were free to proceed to the next site.

We computed the optimal strategy for each session type following the prey selection model of foraging theory [4]. Each feeder location was modeled as a prey type with a handling time equal to its delay. Given these parameters, we found that the rate-maximizing strategy in all session types was to wait for food at only the shortest-delayed site. Our subjects did not employ this strategy. Instead, rats nearly always accepted both short and medium length delays, and often waited for even the longest delay.

We tested several possible explanations for rats’ behavior on this task, including satisficing [5], operant matching [6] and integration over shorter temporal horizons. We also ran a reinforcement learning [7] model of the task over a range of discounting rates and action selection parameters. None of these matched the observed behavior.

The sunk cost effect predicts that as investment in a option increases, willingness to abandon that option decreases. Running between feeder locations entails an energetic investment that could bias subjects towards waiting out long delays despite the resulting decrease in reward rate. We fit an aversion parameter *A* to model subjects’ reluctance to skip feeder sites. Subjects’ unwillingness to abandon sites varied across session types, indicating energetic expenditure (which is equal across sessions) cannot fully account for their suboptimal behavior. Interestingly, we found that *A* correlated positively with opportunity cost. These data suggest that rats’ decisions are influenced by sunk costs, and that the investment they track likely incorporates both energetic costs and the reinforcement statistics of the environment.
